# Clinical and microbiological characteristics of tigecycline non-susceptible *Klebsiella pneumoniae* bacteremia in Taiwan

**DOI:** 10.1186/1471-2334-14-1

**Published:** 2014-01-01

**Authors:** Yi-Tsung Lin, Fu-Der Wang, Yu-Jiun Chan, Yung-Chieh Fu, Chang-Phone Fung

**Affiliations:** 1 Division of Infectious Disease, Department of Medicine, Taipei Veterans General Hospital, No. 201, Sec. 2, Shih-Pai Road, Taipei, 112, Taiwan; 2 School of Medicine, National Yang-Ming University, Taipei, Taiwan; 3 Division of Microbiology, Department of Pathology and Laboratory Medicine, Taipei Veterans General Hospital, Taipei, Taiwan

**Keywords:** Antimicrobial resistance, Bacteremia, Clinical characteristics, *Klebsiella pneumoniae*, Tigecycline

## Abstract

**Background:**

Resistance among *Klebsiella pneumoniae* to most antibiotics is on the rise. Tigecycline has been considered as one of the few therapeutic options available to treat multidrug-resistant bacteria. We investigated the clinical and microbiological characteristics of tigecycline non-susceptible *K. pneumoniae* bacteremia.

**Methods:**

Adult patients with tigecycline non-susceptible *K. pneumoniae* bacteremia at a medical center in Taiwan over a 3-year period were enrolled. *K. pneumoniae* isolates were identified by the E-test using criteria set by the US Food and Drug Administration (FDA). Data on the clinical features of patients were collected from medical records. Genes for β-lactamases, antimicrobial susceptibilities and pulsed-field gel electrophoresis (PFGE) results were determined for all isolates.

**Results:**

Of 36 patients, 27 had nosocomial bacteremia. Overall 28-day mortality was 38.9%. The MIC_50_ and MIC_90_ of tigecycline were 6 and 8 mg/L, respectively. No carbapenemase was detected among the 36 isolates. Twenty isolates carried extended spectrum β-lactamases and/or DHA-1 genes. No major cluster of isolates was found among the 36 isolates by PFGE. Intensive care unit onset of tigecycline non-susceptible *Klebsiella pneumoniae* bacteremia was the only independent risk factor for 28-day mortality.

**Conclusions:**

The high mortality of patients with tigecycline non-susceptible *K. pneumoniae* bacteremia may suggest a critical problem. Further study to identify the possible risk factors for its development and further investigation of this type of bacteremia is necessary.

## Background

*Klebsiella pneumoniae* is a common cause of community-acquired and hospital-acquired Gram-negative infection of the bloodstream [1]. The vast majority of *K. pneumoniae* infections (including urinary-tract infections, pneumonia, and intra-abdominal infections) are associated with hospitalization [2]. In Taiwan, *K. pneumoniae* is also a major cause of community-acquired pyogenic infection [3-7].

*K. pneumoniae* strains harboring extended spectrum β-lactamases (ESBL) and, more recently, carbapenemase that confer resistance to multiple antibiotics, have been described in many parts of the world. These multidrug-resistant (MDR) organisms affect the choice of antimicrobial therapy, and are a major cause for increasing hospital costs and duration of hospitalizations [8].

Tigecycline is an expanded broad-spectrum antibiotic representing a new class called “glycylcyclines”. In comparison with other tetracyclines, tigecycline displays higher *in vitro* activity against several Gram-positive and various Gram-negative microorganisms (including MDR strains). Resistance among *K. pneumoniae* to most antibiotics is on the rise globally [9]. However, tigecycline remains active against many strains, and regarded as one of the few therapeutic options available for treating MDR bacteria.

Tigecycline resistance in *K. pneumoniae* has increasingly been reported in European countries, with a non-susceptible prevalence of 7.5–50% [10]. Reports from North America, South America and Asia have demonstrated that the non-susceptible prevalence is <10% [10]. The increasing prevalence of tigecycline resistance is a growing concern clinically. However, reports of patients infected with tigecycline- resistant *K. pneumoniae* have rarely been identified [11-14]. Furthermore, no study has systematically analyzed clinical data on tigecycline non-susceptible isolates causing bloodstream infection in adult patients.

We investigated the clinical and microbiological characteristics of bacteremia caused by tigecycline non-susceptible *K. pneumoniae* in adult patients during 2010–2012 from a medical center in Taiwan.

## Methods

### Study population

This retrospective study was conducted at Taipei Veterans General Hospital (a 2900-bed tertiary-care teaching hospital) from January 2010 to December 2012. The clinical and microbiological data of all consecutive patients with ≥1 positive blood culture for *K. pneumoniae* strains showing non-susceptibility to tigecycline (minimum inhibitory concentration (MIC) > 2 mg/L) were collected. For patients with more than 2 positive blood cultures, only the first blood culture was included. Patients <20 years of age and those with incomplete medical records were excluded. All clinical isolates were taken as part of standard care. The study protocol was approved by the Review Board of Taipei Veterans General Hospital (Taipei, Taiwan).

### Strain identification and antimicrobial susceptibility test

Identification and antimicrobial susceptibility of *K. pneumoniae* were determined using the Vitek2 system (bioMérieux, Marcy-l’Etoile, France). Antimicrobial susceptibility was interpreted according to the guidelines of the Clinical and Laboratory Standards Institute (CLSI) [15]. *K. pneumoniae* strains showing non-susceptibility to tigecycline (MIC >2 mg/L) according to the Vitek2 system were tested further by the E-test method (AB Biodisk, Solna, Sweden) according to manufacturer instructions. Breakpoints for tigecycline were those set by the US Food and Drug Administration (FDA) (2 and 8 mg/L for susceptible and resistant, respectively) [16]. *Escherichia coli* ATCC 25922, *E. coli* ATCC 35218 and *Pseudomonas aeruginosa* ATCC 27853 were used as control strains in the susceptibility assays.

### Molecular characterization of β-lactamases

All tigecycline non-susceptible strains were tested by polymerase chain reaction (PCR) amplification and DNA sequencing for various carbapenemase genes (encoding class-B families IMP, VIM, NDM-1, GIM, SPM and SIM; class-A families NMC, IMI, SME, KPC and GES; and class-D family OXA-48), plasmid-borne ampC-like genes (encoding CMY, DHA and ACT) and ESBL genes (encoding CTX-M, TEM, and SHV) using methodology and primers described previously [17].

### Capsular genotype

To determine the capsular genotypes of *K. pneumoniae*, we undertook *cps* genotyping by the PCR detection of K serotype-specific alleles at *wzy* and *wzx* loci, including serotypes K1, K2, K5, K20, K54, and K57, as described previously [18]. These capsular serotypes are regarded as having the highest association with an invasive disease or pathogenicity [19].

### Pulsed-field gel electrophoresis (PFGE)

Total DNA was prepared and PFGE was conducted as described previously [17]. Restriction enzyme XbaI (New England BioLabs, Ipswich, MA, USA) was used at the temperature suggested by the manufacturer. Restriction fragments were separated by PFGE in 1% agarose gel (Bio-Rad, Hercules, CA, USA) in 0.56 TBE buffer (45 mM Tris, 45 mM boric acid, 1.0 mM ethylenediamine tetra-acetic acid (EDTA); pH 8.0) for 22 h at 200 V and 14°C and with ramp times of 2–40 s using CHEF Mapper apparatus (Bio-Rad). Gels were then stained with ethidium bromide and photographed under ultraviolet light. The Dice coefficient was used to calculate similarities. The unweighted pair-group method with arithmetic mean (UPGMA) was used for cluster analyses by BioNumerics ver5.10 (Applied Maths, Austin, TX, USA).

### Data collection

Medical records were reviewed to extract pertinent information, including demographic characteristics; comorbid conditions and the Charlson Comorbidity Index [20], duration of hospital stay, duration of therapy with individual antimicrobial drugs, antimicrobial therapy administrated before the onset of bacteremia, and a ventilator, central venous catheter, or a Foley catheter at the time of bacteremia onset. The onset of bacteremia was defined as the day the blood culture that eventually yielded *K. pneumoniae* was obtained. Bacteremia was defined as nosocomial-acquired if the index blood culture was collected >48 h after hospital admission and no signs or symptoms of infection was noted upon hospital admission. Infections with onset ≤48 h after hospital admission were classified as healthcare-associated or community-acquired in accordance with definitions described previously [6]. Episodes of bacteremia were considered acquired in the intensive care unit (ICU) if they appeared >48 h after ICU admission. Infection severity was evaluated using the Acute Physiology and Chronic Health Evaluation (APACHE) II score and Pitt Bacteremia Score described within 24 h before the onset of bacteremia [21,22]. Appropriate antimicrobial therapy was defined as administration of ≥1 antimicrobial agent to which the causative pathogen was susceptible *in vitro* within 48 h after the onset of bacteremia with an approved route and dose appropriate for end-organ function. Antimicrobial therapy that did not meet this definition was considered inappropriate. The primary outcome measure was all-cause 28-day mortality after the onset of *K. pneumoniae* bacteremia.

### Statistical analyses

Categorical variables are absolute numbers and their relative frequencies. Quantitative variables are mean and standard deviation (SD) if distributed normally, or as median and interquartile range (IQR) if distributed non-normally. Contingency data were analyzed by the two-tailed chi-square test or the Fisher’s exact test. Continuous data were analyzed by the Student’s *t*-test or the Mann–Whitney U test. An exact logistic regression was used in the multivariate analyses to analyze risk factors for 28-day mortality. All factors with p < 0.1 in the univariate analyses were included in the exact logistic regression. p < 0.05 was considered as significant. Statistical analyses were carried out using SAS ver9.2 (SAS Institute Inc., Cary, NC, USA).

## Results

### Microbiological characteristics

During the study period, 40 patients with bacteremia due to tigecycline non-susceptible *K. pneumoniae* were identified. Thirty-six patients were finally enrolled after confirmation of susceptibility by the E-test method. The MIC_50_ and the MIC_90_ of tigecycline were 6 and 8 mg/L, respectively. The tigecycline non-susceptibility rate for *K. pneumoniae* bacteremia during the study period was 0.05%. The *in vitro* activities of the tested antimicrobial agents against the 36 tigecycline non-susceptible *K. pneumoniae* isolates are shown in Table 1. Among the 36 isolates, 8 isolates were not susceptible to ertapenem (MIC ≥1 μg/mL). Of the 8 isolates not susceptible to ertapenem, 5 isolates were resistant to ertapenem (MIC ≥2 μg/mL) and two of them were not susceptible to imipenem (MIC = 2 and 4 μg/mL, respectively).

**Table 1:** ***In vitro *** **activities of tested antimicrobial agents against 36 tigecycline non-susecptible *K. pneumoniae *** **isolates**

**Antimicrobial agent**	**MIC**^**a **^**range (μg/mL)**	**MIC**_**50**_^**b **^**(μg/mL)**	**MIC**_**90**_^**c **^**(μg/mL)**	**No. (%) of isolates susceptible**
Tigecycline	3-32	6	8	
Ciprofloxacin	<0.25 to >4	2	>4	15 (42)
Levofloxacin	0.5 to >8	>8	>8	14 (39)
Ampicillin-sulbactam	8 to >32	>32	>32	2 (6)
Piperacillin-tazobactam^*^	8 to >128	32	>128	12 (48)
Cefazolin	<1 to >64	>64	>64	13 (36)
Ceftriaxone	<1 to >64	<1	>64	21 (58)
Ceftazidime	<1 to >64	16	>64	16 (44)
Cefepime	<1 to >64	<1	>64	23 (64)
Amikacin	<2 to >64	<2	16	33 (92)
Gentamicin	<1 to >16	<1	>16	22 (61)
Ertapenem	<0.5 to >8	<0.5	4	28 (78)
Imipenem	<0.25 to 4	<1	<1	34 (94)

^a^MIC: minimal inhibitory concentration.

^b^MIC_50_: MIC for 50% of isolates.

^c^MIC_90_: MIC for 90% of isolates.

^*^Total number is 25 because 11 patients did not undergo the MIC test.

**Figure 1:**
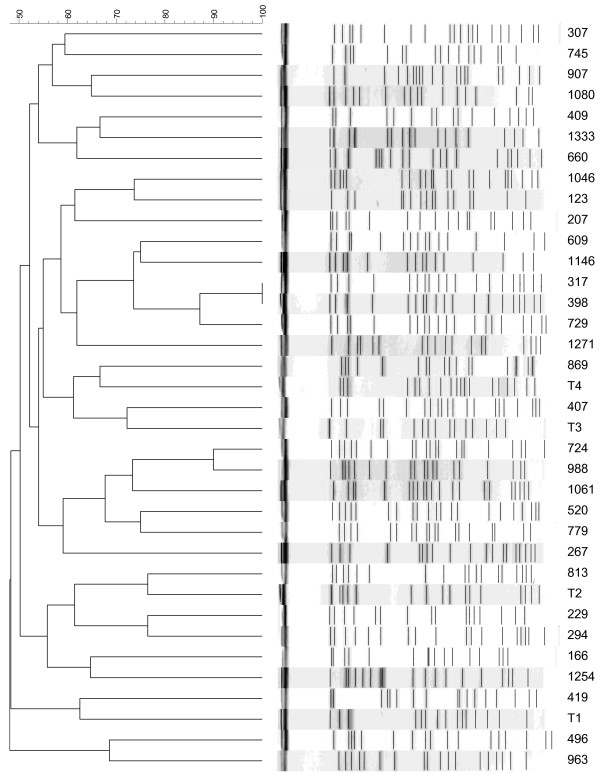
**Pulse-field gel electrophoresis dendrogram of 36** ***K. pneumoniae *isolates.** Most of the isolates did not have a clonal relationship. Isolates 317 and 398 showed identical patterns.

One pair of *K. pneumoniae* strains had indistinguishable PFGE patterns (Figure 1). According to criteria described previously, no major cluster of isolates was found among those 36 isolates.

Carbapenemase genes were not detected among any of the 36 isolates. Among the isolates producing ESBLs, *bla*_CTX-M_ was detected in 6 isolates. Five isolates expressed CTX-M-15, and 1 isolate expressed CTX-M-14. *bla*_SHV-11_, *bla*_SHV-12_ and *bla*_SHV-31_ were detected in 7, 3, and 2 isolates, respectively. With regard to AmpC-type β-lactamases, *bla*_DHA-1_ was detected in 18 isolates, and *bla*_CMY-2_ and *bla*_ACT-1_ were not detected among these isolates (Table 2).

**Table 2:** **Molecular characteristics of the 22 tigecycline non-susceptible *K. pneumoniae *** **isolates with ceftriaxone or ceftazidime (MIC >1 mg/L)**

**Isolate**	**Tigecycline MIC**	**Ertapenem MIC**	**TEM**	**SHV**	**CTX-M**	**DHA-1**
1	3	<0.5	—	SHV-1	—	+
2	3	<0.5	—	SHV-12	—	+
3	3	8	—	-^a^	—	—
4	3	<0.5	TEM-1	SHV-11	—	—
5	4	<0.5	TEM-1	SHV-11	CTXM-15	+
6	4	2	—	SHV-1	CTXM-15	+
7	4	<0.5	—	SHV-12	—	+
8	4	<0.5	—	SHV-31	—	+
9	4	<0.5	TEM-1	SHV-2	—	—
10	4	1	TEM-1	SHV-1	CTXM-15	+
11	6	8	—	SHV-2	—	+
12	6	4	—	SHV-1	—	+
13	6	<0.5	TEM-1	SHV-1	CTXM-15	+
14	6	<0.5	TEM-1	SHV-1	CTXM-15	+
15	6	<0.5	TEM-1	SHV-11	—	+
16	6	1	TEM-1	SHV-11	—	+
17	8	1	TEM-1	SHV-11	CTXM-14	+
18	8	<0.5	—	SHV-12	—	+
19	8	<0.5	—	—^a^	—	+
20	8	8	—	SHV-31	—	—
21	16	<0.5	—	SHV-11	—	+
22	32	<0.5	TEM-1	SHV-11	—	+

^a^The result showed OPK-B.

The results of capsular genotyping for the 6 genotypes (K1, K2, K5, K20, K54 and K57) showed that 2 isolates belonged to capsular type K2, and that 1 isolate was capsular type K54. These 3 isolates did not carry ESBL or plasmid-borne ampC-like genes.

### Clinical characteristics

During the study period, no obvious increasing trends or any cluster of tigecycline non-susceptible *K. pneumoniae* bacteremia was identified. There was no epidemiological relationship between the 2 patients whose isolates had indistinguishable patterns on PFGE. The usage of tigecycline was 11.89 defined daily doses per 1000 patient-days. The clinical characteristics of the patients are summarized in Table 3. Of these patients, 27 (75%) acquired nosocomial *K. pneumoniae* bacteremia, and 14 patients (38.9%) acquired *K. pneumoniae* bacteremia in the ICU. Among the 9 patients with community-onset bacteremia, 6 patients (16.7%) were categorized as having healthcare-associated bacteremia. Most patients received antibiotics during the last 30 days. However, only 13 patients (36.1%) received tigecycline. We investigated further the previous culture-positive data for tigecycline-susceptible *K. pneumoniae* and associated tigecycline use in the past 90 days. Nine patients with tigecycline-susceptible *K. pneumoniae* isolates were identified, and 2 patients with tigecycline-susceptible *K. pneumoniae* isolated within 30 days before bacteremia has been treated with tigecycline.

**Table 3:** **Characteristics of 36 adult patients with tigecycline non-susecptible *K. pneumoniae *** **bacteremia**

**Characteristic**	**n (%)**
Age (years, median, IQR)	75.5, 63.5–81.7
Male sex	27 (75)
Underlying disease	
Immunosuppression^a^	9 (25)
Diabetes mellitus	18 (50)
Chronic obstructive lung disease	5 (13.9)
Cerebral vascular disease	10 (27.8)
Chronic kidney disease, stage = 4 or 5	13 (36.1)
Hemodialysis	11 (30.6)
Malignancy	14 (38.9)
Hematological malignancy	3 ( 8.3)
Solid tumor	11 (30.6)
Liver cirrhosis	3 (8.3)
Charlson comorbidity score (median, IQR)	3, 2–6
Community-*acquired infections*	3 ( 8.3)
Healthcare-associated infections^b^	6 (16.7)
Nosocomial infections^b^	27 (75)
ICU	14 (38.9)
Medical ward	8 (22.2)
Surgical ward	5 (13.9)
Polymicrobial infection	2 (5.6)
Antibiotic exposure in the past 30 days, ≥3 days	
Any	29 (80.6)
Tigecycline	13 (36.1)
Glycopeptides	11 (30.6)
β-lactams plus β-lactamase inhibitors	12 (33.3)
First- and second-generation cephalosporins	11 (30.6)
Third- or fourth-generation cephalosporins	10 (27.8)
Carbapenem	9 (25)
Fluoroquinolones	8 (22.2)
Metronidazole	9 (25)
Ward, duration of stay and devices at the time of bacteremia	
Acquired after ICU >48 h	14 (38.9)
Days of hospitalization before bacteremia (median, IQR)	48, 18–82
Urinary catheter	17 (47.2)
Central venous catheter	23 (63.9)
Mechanical ventilation	18 (50)
Pitt bacteremia score (mean ± SD)	4 ± 4
APACHE II score (mean ± SD)	23 ± 10
Infection sources and clinical syndrome	
Pneumonia	10 (27.8)
Intra-abdominal infection other than biliary-tract infection	5 (13.9)
Biliary-tract infection	7 (19.4)
Skin and soft-tissue infection	2 (5.6)
Catheter infection	1 (2.8)
Urinary-tract infection	1 (2.8)
Unknown primary focus	10 (27.8)
Appropriate antibiotic treatment	17 (47.2)
Outcome	
Presentation with septic shock	19 (52.8)
Requiring admission to ICU after bacteremia	19 (52.8)
In-hospital death	17 (47.2)
28-day mortality	14 (38.9)

IQR, interquartile range; ICU, intensive care unit; APACHE, Acute Physiology and Chronic Health Evaluation; SD, standard deviation.

^a^Immunosuppression includes patients who underwent solid-organ transplantation, corticosteroid therapy, and immunosuppression therapy.

^b^Bacteremia was defined to be nosocomial-acquired if the index blood culture was collected >48 h after hospital admission and no signs or symptoms of infection were noted at hospital admission. Bacteremia with onset ≤48 h after hospital admission was classified as healthcare-associated infection if patients met any of the following criteria: having received intravenous therapy at home or in an outpatient clinic during the past 30 days; having received renal dialysis in a hospital or clinic during the past 30 days; having hospitalized for 2 or more days during the past 90 days; or having resided in a nursing home or long-term care facility.

Pneumonia, intra-abdominal infection, and biliary-tract infection (BTI) were the common sources of bacteremia. The clinical syndromes of patients infected with the capsular type-K2 strain were pneumonia and BTI. BTI was evident in the patient infected with the capsular type K54. The treatment regimen after the sensitivity reports were available included: 13 patients received carbapenem, 11 patients received cephalosporins, 4 patients received fluoroquinolones, and 2 patients received β-lactam/β-lactamase inhibitors. The remaining 6 patients died before the sensitivity reports were available. Among these 6 patients, 4 patients received tigecycline, and 2 patients received carbapenem empirically. The overall 28-day mortality among patients infected with tigecycline non-susceptible *K. pneumoniae* was 38.9%.

Univariate analyses of the factors associated with 28-day mortality are shown in Table 4. The exact logistic regression model showed that the only independent risk factor for 28-day mortality is ICU onset of tigecycline non-susceptible *Klebsiella pneumoniae* bacteremia (odds ratio (OR) 13.46, 95% confidence interval (CI) 1.35–637.17, p = 0.021).

**Table 4:** **Risk factors for 28-day mortality among 36 patients infected by tigecycline non-susecptible *K. pneumoniae *** **bacteremia**

	**Survivors (n = 22), n (%)**	**Non-survivors (n = 14), n (%)**	**p**	**Adjustedp**^**a**^
Age (years, median, IQR)	76.5, 69.3–84.3	71, 60.3–79	0.175	
Male sex	17 (77.3)	10 (71.4)	0.988	
Underlying disease				
Immunosuppression^b^	5 (22.7)	4 (28.6)	0.988	
Diabetes mellitus	9 (40.9)	9 (64.3)	0.305	
Chronic obstructive lung disease	2 (9.1)	3 (21.4)	0.574	
Hemodialysis	5 (22.7)	6 (42.9)	0.364	
Malignancy	7 (31.8)	7 (50)	0.458	
Liver cirrhosis	2 ( 9.1)	1 (7.1)	1.000	
Cerebral vascular disease	6 (27.3)	4 (28.6)	1.000	
Charlson comorbidity score (median, IQR)	3, 2–5	4, 3–8.3	0.171	
Pitt bacteremia score (mean ± SD)	3.3 ± 3.1	5.9 ± 3.7	0.037	0.847
APACHE II score (mean ± SD)	20.1 ± 0.6	27.6 ± 11.0	0.016	0.958
Acquired after ICU >48 h	3 (13.6)	11 (78.6)	<0.001	0.021
Infection sources and clinical syndrome				
Pneumonia	3 (13.6)	7 (50)	0.047	0.569
Intra-abdominal infection other than biliary-tract infection	2 (9.1)	3 (21.4)	0.574	
Biliary-tract infection	7 (31.8)	0 (0)		
Unknown primary focus	8 (36.4)	2 (14.3)	0.289	
Presentation with septic shock	9 (40.9)	10 (71.4)	0.147	
Appropriate antibiotic treatment	12 (54.6)	6 (42.9)	0.733	
Ertapenem non-susceptibility	6 (27.3)	2 (14.3)	0.628	
Tigecycline MIC >4 μg/mL	10 (45.5)	8 (57.1)	0.733	
Tigecycline MIC >6 μg/mL	4 (18.2)	6 (42.9)	0.220	
ESBL-producing strain	10 (45.5)	8 (57.1)	0.733	

IQR, interquartile range; ICU, intensive care unit; APACHE, Acute Physiology and Chronic Health Evaluation; SD, standard deviation.

^a^All factors with p < 0.1 in univariate analyses were included in the exact logistic regression.

^b^Immunosuppression includes patients who underwent solid-organ transplantation, corticosteroid therapy, and immunosuppression therapy.

## Discussion

We demonstrated here, for the first time, the clinical and microbiological characteristics of tigecycline non-susceptible *K. pneumoniae* bacteremia in Taiwan. Most patients had nosocomial bacteremia, and the overall 28-day mortality was 38.9%. The MIC_50_ and MIC_90_ of tigecycline were 6 and 8 mg/L, respectively. Among all isolates, 18 were ESBL producers, and DHA-1 was detected in 18 isolates. No carbapenemase genes were detected among all 36 isolates. According to the results of PFGE, most isolates were epidemiologically unrelated.

Interpretive criteria for the *in vitro* susceptibility testing of tigecycline are based on the breakpoints approved by the FDA or on those published by the European Committee on Antimicrobial Susceptibility Testing. The CLSI has not released tentative or approved guidelines. Differences in susceptibility results according to the MIC interpretive criteria of the two guidelines as well as the testing methods used have been emphasized in several reports [23]. One recent study questioned the performance of the Vitek2 automated system to reliably determine the susceptibility of Enterobacteriaceae species other than *E. coli* to tigecycline [23]. Moreover, it has been reported that the E-test shows the best correlation with the broth microdilution method, and exhibits lowest error rates as well as highest essential agreement and categorical agreement [24]. In general, the Vitek2 automated system produces higher MIC values than E-test doses, which can potentially result in false resistance findings using the former approach [24]. E-test susceptibility results obtained using the FDA criteria could be considered as a reliable method for tigecycline testing, as shown previously [25]. In the current study, isolates reported as being non-susceptible to tigecycline by the Vitek2 system were confirmed with the E-test method to avoid the misclassification of the results.

Common sources of bacteremia in our study include infections of the lower respiratory tract, BTI, and intra-abdominal infections. One report suggested that clinicians should be aware of the potential risk for treatment-induced tigecycline resistance, especially if urine is the main source of bacteria [22]. In contrast to that report, we identified only 1 case with a urinary-tract infection. Physicians should recognize that the emergence of tigecycline non-susceptible *K. pneumoniae* bacteremia can be associated with various syndromes.

The mechanisms of resistance mediating the effectiveness of tigecycline have been attributed to RNDtype transporters and other efflux pumps [10]. Analyses of clinical studies suggest that long-term tigecycline monotherapy may carry a higher risk for developing tigecycline resistance [10]. One recent study investigating non-susceptibility in *K. pneumoniae* and *E. coli* isolates causing neonatal septicemia found increasing tigecycline MIC values from 2007 to 2010. This increase cannot be attributed to the use of tigecycline because of the restricted use of this antibiotic in their hospital. The author suggested that the elevated MIC of tigecycline might be attributed indirectly to the use of other antibiotics that are also effluxed through the AcrAB–TolC pump [14]. In the current study, although most patients received one or more antibiotics in the past 30 days, only 36.1% of subjects were treated with tigecycline in the past 30 days. Further study to analyze the risk factors for tigecycline non-susceptible *K. pneumoniae* infection is necessary.

In the present study, PFGE analyses revealed that tigecycline non-susceptible *K. pneumoniae* isolates causing bacteremia were not clonal in Taiwan. This implies that the acquisition of tigecycline non-susceptibility in invasive *K. pneumoniae* isolates was most likely through independent mechanisms. Such non-clonal occurrence of tigecycline non-susceptibility in invasive *K. pneumoniae* isolates suggests that tigecycline resistance may be due to selective pressure, including increasing, inappropriate and inadequate use of antibiotics. Antibiotic stewardship will be essential for helping us understand whether this holds true in real-life situations.

The 28-day mortality among patients with tigecycline non-susceptible *K. pneumoniae* bacteremia was 38.9%. The case mortality of *K. pneumoniae* bacteremia has been found to be ≈20% [1]. With respect to drug-resistant *K .pneumoniae* bacteremia, the mortality in those with carbapenem-resistant *K. pneumoniae* bacteremia is ≈40–50% [21,26-28], and ≈20–30% in ESBL-producing *K. pneumoniae* bacteremia [21,22]. The present report is the first to point out high mortality among patients with tigecycline non-susceptible *K. pneumoniae* bacteremia in Taiwan. One recent study found that most (75%) of the tigecycline resistant *K. pneumoniae* were *Klebsiella pneumoniae* carbapenemase producers [29]. Surprisingly, no carbapenemase-producing *K. pneumoniae* were identified in the current study. The emergence of tigecycline non-susceptible *K. pneumoniae* bacteremia, in addition to carbapenem resistance, may become an imminent issue in the foreseeable future. We also found that the ICU onset of tigecycline non-susceptible *K. pneumoniae* bacteremia was an independent risk factor for 28-day mortality. Resistance to tigecycline could be simply a marker of disease severity as shown in infections caused by other resistant microorganisms. In addition, we identified 3 isolates belonging to the capsular serotypes K2 and K54, which are thought to be associated most closely with an invasive disease and/or pathogenicity. The MDR *K. pneumonaie* strains rarely belonged to the virulent capsular types (K1, K2, K5, K20, K54, and K57). It would be of interest to study the relationship between these capsular types and tigecycline non-susceptible strains.

Our study had several limitations. The important limitations were inherent to its retrospective design and the limited number of occurrence in a single institution. Caution must be taken to interpret data from a small number of cases. In addition, our study did not include a control group to identify specific risk factors or to compare the prognostic implications. Finally, we did not investigate the possible mechanism (e.g., overexpression of different efflux pumps) among these *K. pneumoniae* isolates. Nevertheless, our study was the first to describe the characteristics of tigecycline non-susceptible *K. pneumoniae* bacteremia in Taiwan, and will provide an important foundation for further work in this emerging area of interest.

## Conclusion

The high mortality of patients with tigecycline non-susceptible *K. pneumoniae* bacteremia may indicate a critical clinical problem. The emergence of tigecycline non-susceptible *K. pneumoniae* bacteremia in our hospital merits further studies to identify potential risk factors for its development.

## Abbreviations

BTI: Biliary-tract infection; CLSI: Clinical and laboratory standards institute; ESBL: Extended-spectrum β-lactamases; PFGE: Pulsed-field gel electrophoresis; FDA: Food and drug administration.

## Competing interests

The authors declare that they have no competing interests.

## Authors’ contributions

YTL conceived the study, and participated in its design and coordination. YTL, FDW, YJC, and YCF reviewed and collected the data. YTL analyzed and interpreted the data. YTL drafted the manuscript. FDW and CPF reviewed the manuscript. All authors approved the final manuscript.

## Pre-publication history

The pre-publication history for this paper can be accessed here:

http://www.biomedcentral.com/1471-2334/14/1/prepub
